# Concurrent and lagged physiological synchrony during mother–child interaction and their relationship to positive affect in 8- to 10-year-old children

**DOI:** 10.1038/s41598-023-43847-8

**Published:** 2023-10-18

**Authors:** Yasemin Zehra Capraz, Kerstin Konrad, Vanessa Reindl

**Affiliations:** 1 Child Neuropsychology Section, Department of Child and Adolescent Psychiatry, Psychosomatics and Psychotherapy, Medical Faculty, RWTH Aachen University, Aachen, Germany; 2 JARA-Brain Institute II, Molecular Neuroscience and Neuroimaging, RWTH Aachen & Research Centre Juelich, Juelich, Germany; 3 Psychology, School of Social Sciences, Nanyang Technological University, Singapore, S639818 Republic of Singapore

**Keywords:** Psychology, Psychology and behaviour, Biomarkers

## Abstract

Mother–child interaction has been characterized by a fine-tuning of behavior and physiological activity. Yet, little is known about the dynamics of mother–child physiological synchrony during early school age and their associations to positive affect. To investigate these processes, 42 mother–child dyads, with children aged 8 to 10 years, played an interactive game while their interbeat intervals (IBI) and respiratory sinus arrhythmia (RSA) were measured simultaneously. IBI/RSA synchrony was calculated using cross-correlations of the IBI/RSA second-by-second time series for lags − 3 to + 3 seconds. Mother’s and child’s individual and shared positive affect were microcoded. During the interactive tasks, IBI and RSA synchrony significantly increased compared to control conditions. RSA and affect synchrony were significantly stronger for negative compared to positive lags indicating a stronger child leads/mother follows covariation. Further, dyad’s IBI and RSA synchrony were significantly associated to mother’s and child’s individual positive affect. Our data suggest that in low-risk community samples, mothers may respond to their children’s positive affect by matching their own affect and physiology. Investigating these temporally precise, concurrent and lagged synchrony processes may open up new avenues for understanding the ways in which parent–child interactions contribute to child developmental outcomes.

## Introduction

Parent–child synchrony is reflected in the coordination of behavioral and physiological rhythms that ultimately forms a “relational unit”, providing the basis for the child’s later social-emotional development^1^. Synchrony can broadly be defined as a “nonrandom, patterned, temporal covariation of the timing or form of behaviors, internal states, or events”^2^. It is therefore not an unchanging steady state, but rather an interactive back and forth through modulation of behavior, affect and physiology in relation to changes in environmental conditions (see also^3,4^). In general, dyadic synchrony can be explored at different levels of analysis^5^: While physiological synchrony describes the coregulation of neural, hormonal and autonomic nervous system (ANS) activity, behavioral synchrony may be characterized e.g., by a coordination of body movements, gaze, attention and affect between parent and child during a particular, mostly interactive, context.

Furthermore, with the application of concurrent recordings in two interacting partners, it is possible to distinguish between different forms of temporal relationships underlying reciprocal interactions, allowing more refined inferences about how the parent’s behavior and physiology are related to the behavior and physiology of the child. Two different temporal relationships can be considered here, concurrent and time-lagged synchrony. While the former describes parent’s and child’s covariations in physiological or behavioral responses occurring at the exact same point in time, the latter describes sequential changes in physiological/behavioral responses of the interaction partners (i.e., changes in mother associated with subsequent changes in child or vice versa)^2^ and may thus indicate potential directionality^6^. Further, signals may be positively or negatively related to each other, either concurrently or lagged, indicating a positive or negative co-regulation of physiological processes^7^.

In the beginning of life, regulatory processes are almost entirely dependent upon parents, so synchronizing behavior during this period is usually considered as an indicator of a parent reading the baby’s cues accurately (infant-leads-mother-follows play^8^). However, later during development the child becomes more and more an active interaction partner in the dyad, thus, synchrony measures might then reflect a coordinated exchange between the parent and the child^9^. Nevertheless, past research often focuses on infancy and early childhood whereas little is known on the dynamics and interdependence of physiological and behavioral synchrony during early school age. During this period of time, in which children’s social context changes and their independence grows, their abilities to self-regulate affect, behavior, and attention continues to develop^10^. The study of these processes in parents and their school-aged children might therefore contribute to a better understanding of the biological mechanism of adaptive parent–child interactions and highlight ways in which child-parent relationships could be leveraged as a mechanism to prevent maladaptive developmental outcomes.

### Relationship between affective and physiological synchrony

Although parent–child biobehavioral synchrony is considered to provide an important foundation for children’s development^1^, still few studies addressed biobehavioral synchrony during parent–child interactions across multiple levels of analysis. Positive emotions are known for their key role in fostering both mental and physical well-being, facilitating social connectedness and strengthening the resilience to stress^11–15^. As such, the way in which parent and child respond to and coordinate their positive affect may have important implications for child development (e.g.^16^). For instance, in a study examining emotional expressions in mothers and their typically developing adolescent girls, interdyad differences in moment-to-moment dynamics of positive emotions were associated with adolescents’ social anxiety symptoms^17^. Given the effects of different emotions on peripheral physiological responses, synchrony in positive affect may also be associated with increased ANS synchrony.

In line thereof, increases in interbeat interval (IBI) synchrony were found in mother-infant dyads during episodes of affect synchrony (the proportion of time mother and infant matched their positive affect) and vocal synchrony (the proportion of time they emitted positive vocalizations simultaneously)^18^. However no significant increases in IBI synchrony were observed in episodes of gaze synchrony or in nonsynchronous episodes of the dyad as indicated by bootstrapping analysis. Furthermore, in mother–preschooler dyads, more maternal teaching behavior, e.g., asking questions or giving instructions, was associated with higher respiratory sinus arrhythmia (RSA) synchrony over time, while greater maternal disengagement, e.g., a lack of interaction, including ignoring child’s bids for attention, was associated with weaker synchrony over time (using autoregressive models with maternal teaching/disengagement as between-subjects predictor^19^). While these studies point towards positive associations between physiological and affective or behavioral synchrony, Suveg et al. showed that positive behavioral synchrony was negatively correlated with physiological synchrony in cases of high family risk but not significantly correlated in cases of low family risk in mother-preschooler dyads^9^. In their study, positive behavioral synchrony reflected the dyad’s ability to work together effectively and a generally positive tone of the interaction. Other studies have examined group differences between healthy controls and dyads with specific psychopathological conditions or risk factors, such as maternal depression^20^, child’s internalizing^21^ or externalizing problems^7^, and a recent meta-analysis reported less mother–child RSA synchrony in high-risk samples^22^. Although differences between groups in behavioral, and in particular affective, synchrony are likely (e.g., maternal history of depression has been associated with reduced mother–child concurrent and lagged synchrony in positive facial affect^23^), the direct links between physiological and affective synchrony in typically developing children, particularly in early school age, are mostly unknown.

### Measuring physiological synchrony

The ANS consists primarily of the sympathetic as well as the parasympathetic nervous system which are responsible for dynamic regulations of internal visceral organs including cardiac, respiratory and glandular systems in response to endogenous and exogenous environmental conditions^24^. A well-studied measure is the IBI, a temporal parameter that describes the time between two successive heartbeats and decreases with increasing heart rate^25^. It can be assessed on a moment-to-moment basis thus allowing a detailed analysis of patterns of physiological states; however, it is reflective of the activity of both sympathetic as well as parasympathetic nervous system, making it difficult to differentiate their relative contributions^5^. By contrast, RSA, which describes rhythmic fluctuations in heart rate at the respiration frequency, is considered one of the most selective noninvasive indices of parasympathetic activity^26^. According to the Polyvagal Theory, the parasympathetic regulation increases in calm and non-stressful contexts, promoting rest-and-repair functions and supporting social engagement^27^. In restful periods, the vagal influence acts as a “brake” on sympathetic activation and promotes a steady metabolism. When situational stressors arise, this vagal regulation is suppressed or withdrawn, and resources are mobilized to overcome challenges. In line thereof, it has been shown that lower levels of baseline RSA or deficits in vagal regulation are associated with deficits in social communication and engagement behaviour as well as maladaptive stress response^27,28^, while higher levels of RSA withdrawal are associated with better emotional regulation and coping abilities^29,30^. As such, this parameter may help to assess physiological states during social engagement in resting and challenging situations. Although previous research demonstrated that RSA changes rapidly in response to environmental change, still many traditional methods to analyse RSA rely on estimates aggregated across longer time intervals (e.g., 30 s intervals)^6,31^. More recently, however, novel approaches using Peak Matched Multiple Windows have been validated that allow analyses of time series of second-by-second RSA data providing new insights into the fine-tuned RSA dynamics in interacting partners^32^. Importantly, analysing synchrony across different physiological systems, e.g., IBI and RSA, may allow a more nuanced understanding of the joint and individual contributions of sympathetic and parasympathetic activity to dyadic regulatory processes of parent and child.

### The current study

The present study aims to explore concurrent and time-lagged synchrony in low-risk mother–child dyads during an interactive task and how this is related to the dyad’s positive affect. Mother–child dyads played two rounds of a labyrinth game together (‘La1’ and ‘La2’) in which they had to jointly navigate a marble through a tilting maze without talking to each other. Thus, in order to successfully perform the task, mother and child had to coordinate their movements nonverbally to achieve a shared goal. To this end, they had to be attuned to each other, perceive each other’s signals and respond accordingly. Of note is that dyads generally enjoyed playing the labyrinth game and showed very little to no negative affect. Thus, our current analyses focused on positive affect only. Dyadic synchrony was measured in IBI and RSA using concurrent and lagged synchrony indices and were compared to the dyadic synchrony when playing the game alone (‘Single’) and when watching a relaxing video together (‘Rest’). During the interactive games (La1 and La2), mother’s and child’s affect were micro-coded. Our primary research questions were to investigate (i) how IBI/RSA synchrony, both concurrent and lagged, are affected by different experimental conditions (Research Question 1), and (ii) how these are linked to mother’s and child’s individual and shared positive affect (Research Question 2). Oftentimes, relationships between physiological and behavioral synchrony measures are analysed across dyads, e.g., correlating task-averaged synchrony values with each other (e.g.^9^), or indirectly by examining differences between groups with certain risk factors (e.g., mothers with and without a history of depression^20^). Here, we extend this analysis by examining the relationships within dyads. While the first type of analysis examines whether more positive affect is associated with higher or lower physiological synchrony across dyads, the second type of analysis asks whether affect-related changes in mother’s and child’s IBI and RSA contribute to increased IBI/RSA synchrony of the dyad. We hypothesized that during the interactive labyrinth task (La1 and La2), IBI and RSA synchrony would be increased compared to Single and Rest. Since parent–child synchrony may become more and more bidirectional from infancy to childhood, we expect to find significant time-lagged synchrony in both directions, from mother to child and from child to mother. Based on Feldman et al., we expected positive affect to be positively associated with IBI / RSA synchrony within the dyad^18^.

## Results

Descriptive results for mean IBI, RSA and affect synchrony as well as mother’s and child’s individual positive affect can be found in Table 1 and SI, Table S1 (*M*, *SD* and range). Microcoded affect from the video recordings served as our measure of affect synchrony. Of note is that no significant correlations were found between microcoded affect and global self-report ratings of emotional valence and arousal after the task. Further, the course of play, i.e., number of successful rounds and times the ball fell into the hole, was coded as a measure of task performance. No significant differences in task performance were found between La1 and La2, indicating no significant behavioral learning effects (see SI, Table S2a,b).

**Table 1:** Means, standard deviations and bivariate correlations for IBI synchrony, RSA synchrony, shared and individual positive affect at lag 0.

Variable		*n*	*M*	*SD*	1	2	3	4	5	6	7	8	9	10	11	12	13	14
IBI synchrony	1. La1	34	0.095	0.071														
	2. La2	37	0.099	0.061	− 0.265													
	3. Single	33	0.002	0.073	− 0.242	0.158												
	4. Rest	39	0.017	0.052	0.002	0.315	0.361*											
RSA synchrony	5. La1	34	0.084	0.171	− 0.007	− 0.296	− 0.291	− 0.125										
	6. La2	37	0.094	0.187	− 0.019	− 0.064	− 0.022	0.006	0.294									
	7. Single	33	− 0.037	0.146	− 0.297	0.023	0.397*	− 0.013	− 0.114	0.280								
	8. Rest	39	0.023	0.180	− 0.057	− 0.074	0.100	0.032	− 0.148	− 0.083	0.266							
Shared pos. affect	9. La1	41	0.071	0.067	0.066	0.092	–	–	− 0.331	− 0.139	–	–						
	10. La2	41	0.041	0.044	0.146	− 0.001	–	–	− 0.179	− 0.052	–	–	0.674**					
Mother pos. affect	11. La1	41	0.135	0.103	0.205	− 0.123	–	–	− 0.332	− 0.189	–	–	0.845**	0.588**				
	12. La2	41	0.090	0.074	0.192	− 0.238	–	–	− 0.303	− 0.161	–	–	0.552**	0.793**	0.670**			
Child pos. affect	13. La1	41	0.122	0.095	− 0.127	0.083	–	–	− 0.238	− 0.200	–	–	0.848**	0.614**	0.595**	0.434**		
	14. La3	41	0.079	0.073	0.005	0.102	–	–	− 0.223	− 0.011	–	–	0.663**	0.926**	0.497**	0.670**	0.657**	
Mother age		42	40.81	4.67	0.511**	− 0.266	0.166	− 0.266	0.032	− 0.099	− 0.109	0.071	− 0.144	0.037	0.014	0.169	− 0.218	− 0.070
Child age		42	9.00	1.25	0.306	− 0.265	− 0.009	− 0.017	− 0.034	− 0.172	− 0.035	0.081	0.035	0.199	0.229	0.325*	− 0.047	0.086

*IBI* interbeat interval, *RSA* respiratory sinus arrhythmia, *Pos* positive affect, *La1* first labyrinth game, *La2* second labyrinth game, *Single* single labyrinth game, *Rest* rest video.

*p < 0.05 (uncorrected), **p < 0.01 (uncorrected).

To examine whether synchrony was observed in IBI, RSA and affect, we first compared the mother–child dyadic synchrony to the synchrony of shuffled adult–child pairs, who participated in the same tasks but independently of each other. This controlled for random similarities in the signals not related to the dyadic interaction. Second, we investigated whether the dyadic synchrony was higher during the interactive (La1, La2) compared to the non-interactive tasks (Rest, Single) by directly comparing the conditions. All analyses were conducted for lags − 3 s (child leads) to lags + 3 s (mother leads) at 1 s-increments. Finally, to examine whether mother or child led the covariation, lag − 1/− 2/− 3 were compared to lag + 1/+ 2/+ 3 synchrony values.

### IBI synchrony

**Figure 1:**
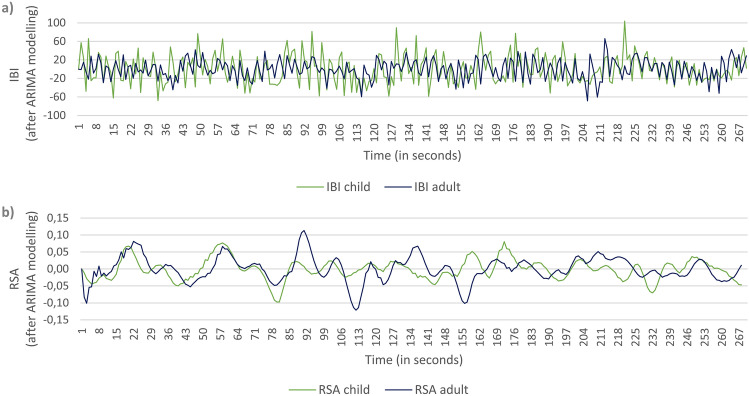
Exemplary illustration of high IBI (**a**) and RSA (**b**) synchrony for one mother–child dyad.

Figure 1 shows an example of two mother–child dyads with high IBI and RSA synchrony at lag 0, respectively.

**Figure 2:**
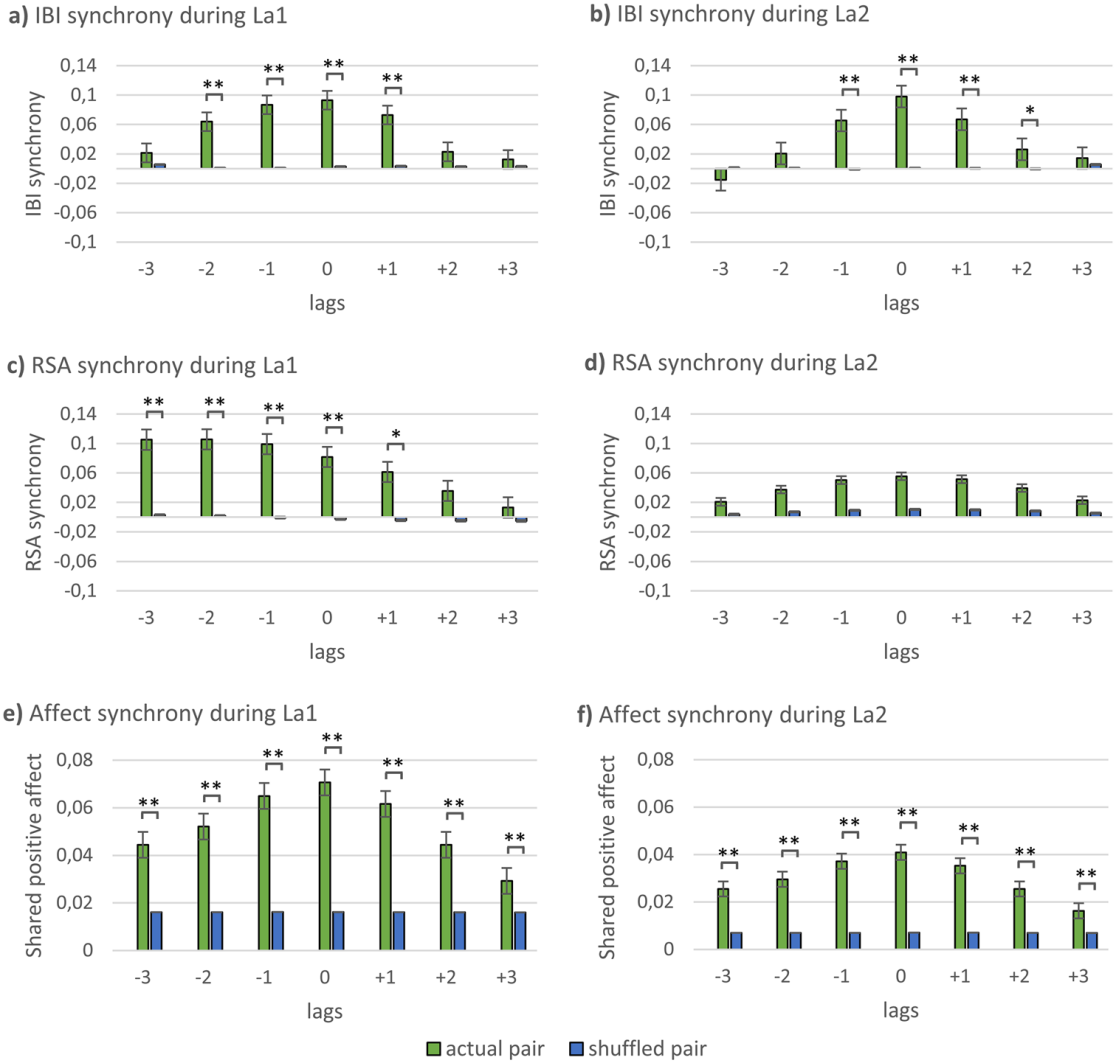
Comparison of actual (green) and shuffled (blue) pairs for IBI (**a**, **b**), RSA (**c**, **d**) and positive affect synchrony (**e**, **f**) during the first and second labyrinth game. *p < 0.05 (corrected), **p < 0.01 (corrected).

Compared to shuffled pairs, mother–child dyads had an increased IBI synchrony during La1 at lags 0, + 1, − 1 and − 2 but not at lags + 2, + 3 and − 3. Further, during La2 they showed an increased synchrony at lags 0, + 1, + 2 and − 1, while no significantly increased IBI synchrony was found at lags + 3 and − 2, − 3. No significant differences were found between actual and shuffled pairs for the two control conditions at none of the lags (see Fig. 2, SI, Table S3a,b).

In our linear mixed models, we found a significant task effect for IBI synchrony for lag 0 (*F*(3) = 22.96; *p*_*adj*_ = 0.002, *R*^2^ = 0.314), lag + 1 (*F*(3) = 13.29; *p*_*adj*_ = 0.002, *R*^2^ = 0.217), lag − 1 (*F*(3) = 20.86, *p*_*adj*_ = 0.002, *R*^2^ = 0.304) and lag − 2 (*F*(3) = 5.41, *p*_*adj*_ = 0.002, *R*^2^ = 0.102), whereas no significant task effect was found for lag + 2, + 3 and − 3 (see SI, Table S4). Post-hoc tests showed that mother–child dyads had a higher synchrony during La1 compared to Single at lag 0, + 1, − 1 and − 2 and compared to Rest at lag 0, + 1, − 1 and − 2. Further, they had a higher synchrony at La2 compared to Single at lag 0, + 1 and − 1 and compared to Rest at lag 0, + 1 and − 1 (see SI, Table S5a–d). Synchrony values did not significantly differ between La1 and La2 (expect for IBI synchrony at lag − 2; see SI, Table S5e). Further, no significant differences were found between negative and positive lags after FDR-correction of *p*-values. To summarize, mother–child dyads showed an increased IBI synchrony during La1 and La2 compared to shuffled dyads for concurrent as well as lagged synchrony.

### RSA synchrony

In line with the results for IBI, actual mother–child dyads showed higher RSA synchrony than shuffled pairs during La1 at lags 0, + 1, − 1, − 2 and − 3. There were no significant differences between actual and shuffled pairs for La1 at lags + 2, + 3 as well as for La2 and the two control conditions at any of the lags (see Fig. 2, SI, Table S3a,b).

In our linear mixed models, a significant task effect was found for lag 0 (*F*(3) = 3.35; *p*_*adj*_ = 0.039, *R*^2^ = 0.064), lag − 1 (*F*(3) = 4.70, *p*_*adj*_ = 0.009, *R*^2^ = 0.089), lag − 2 (*F*(3) = 5.43, *p*_*adj*_ = 0.007, *R*^2^ = 0.102) and lag − 3 (*F*(3) = 5.40, *p*_*adj*_ = 0.007, *R*^2^ = 0.101), whereas no significant task effect was found for lags + 1, + 2 and + 3 (see SI, Table S4). Post-hoc tests showed that mother–child dyads had higher RSA synchrony during La1 compared to Single at lag 0, − 1, − 2 and − 3 as well as compared to Rest at lag − 2 and − 3. For La2, significant differences were found compared to Single at lag − 1. No significant differences were found for La2 compared to Rest (see SI, Table S5a–d). Synchrony values did not significantly differ between La1 and La2 (see SI, Table S5e).

Comparing positive and negative lags, significant differences were found for La1, with higher synchrony values for lag − 1 compared to lag + 1 (*t*(33) =  − 2.41, *p*_*adj*_ = 0.033, *d* = 0.23) and for lag − 2 compared to lag + 2 (*t*(33) =  − 2.47, *p*_*adj*_ = 0.033, *d* = 0.43).

Thus, in line with the IBI results, actual mother–child dyads had an increased RSA synchrony during the joint labyrinth game, however only for the first and not for the second game. Further, higher RSA synchrony was found during La1 for negative than for positive lags, indicating a stronger child leads/mother follows covariation during the first game.

### Affect synchrony

Actual mother–child dyads displayed higher affect synchrony than shuffled pairs during La1 and La2 for all positive and negative lags (see Fig. 2, SI, Table S6). Comparing positive to negative lags, a significant difference was found for lag − 2 compared to lag + 2 at La1 (*t*(40) = 2.613, *p*_*adj*_ = 0.026, *d* = 0.15), with higher values for lag − 2 indicating a stronger child leads/mother follows covariation for affect synchrony during the first joint labyrinth game.

### Relationship between ANS and affect synchrony

Correlations between IBI/RSA synchrony, mother’s and child’s individual and shared positive affect at lag 0 are depicted in Table 1. No significant correlations emerged for La1 or La2, indicating that affect synchrony was not associated with higher ANS synchrony across dyads. Further, since both RSA and affect synchrony were higher for lag − 2 (child leads/mother follows) than for lag + 2 (mother leads/child follows) during La1, we examined in an exploratory analysis whether affect synchrony at lag − 2 was associated with IBI/RSA synchrony at lag − 2. No significant correlations emerged neither for IBI (La1: *r* =  − 0.113, *p*_*adj*_ = 0.75; La2: *r* =  − 0.055, *p*_*adj*_ = 0.75) nor for RSA (La1: *r* =  − 0.149, *p*_*adj*_ = 0.82; La2: *r* =  − 0.012, *p*_*adj*_ = 0.94).

In a next step, we examined whether changes in mother’s and child’s affect contributed to increased IBI/RSA synchrony within the dyad. To this end, we regressed out (i) the participant’s individual positive affect and (ii) their shared positive affect from the participant’s individual IBI/RSA time series, partialling out the influence of individual/shared positive affect. Afterwards, IBI/RSA synchrony was recalculated for lags − 3 s (child leads) to + 3 s (mother leads) and the resulting ‘cleaned’ synchrony values were compared to ‘uncleaned’ synchrony.

**Figure 3:**
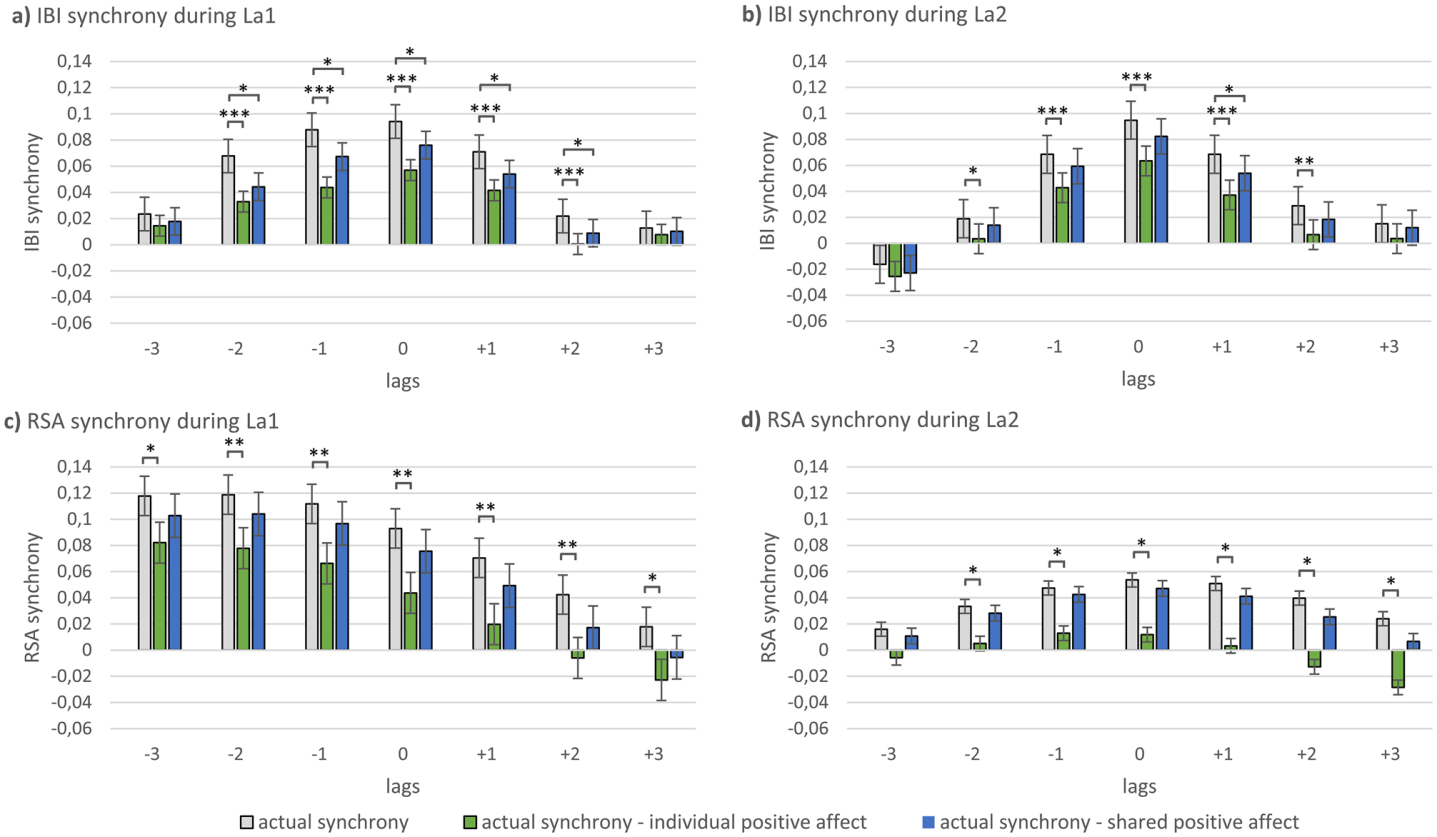
Comparison of actual synchrony (gray) with synchrony after regression of individual affect (green) and synchrony after regression of shared positive affect (blue) for IBI (**a**, **b**) and RSA (**c**, **d**) during the first and second labyrinth game. *p < 0.05 (corrected), **p < 0.01 (corrected), ***p < 0.001 (corrected).

When regressing out *individual* positive affect, a reduced IBI synchrony was observed for both La1 and La2 at lags 0, + 1, + 2, − 1 and − 2 and a reduced RSA synchrony was found for La1 at all lags and for La2 at lags 0, + 1, + 2, + 3, − 1 and − 2 (see Fig. 3, SI, Table S7). To control for the possibility that regression analyses led to reduced synchrony, e.g., by reducing the variability of the IBI/RSA residual time series, results were further compared to shuffled pairs, whereby not the own affect time series but that of other participants were used in the regression. Results were mostly validated by comparison to shuffled pairs, although some results were only marginally significant after FDR correction of *p*-values (see SI, Table S8). Together, these results indicate that regressing out mother’s and child’s *individual* positive affect significantly reduced IBI and RSA synchrony, suggesting that mother’s and child’s positive affect modulated IBI and RSA synchrony during the labyrinth tasks.

When regressing out *shared* positive affect, only IBI synchrony was significantly reduced for La1 at lags 0, + 1, + 2, − 1 and − 2 and for La2 at lag + 1, whereas no significant changes were found for RSA synchrony (see Fig. 3; SI, Table S9). Again, results were mostly validated by comparison to shuffled pairs (see SI, Table S10). Regressing out individual positive affect led to a stronger reduction of IBI and RSA synchrony than regressing out shared positive affect for both La1 and La2 at most of the lags (see SI, Table S11).

## Discussion

As hypothesized, we found increased IBI and RSA synchrony during the joint labyrinth task for concurrent as well as lagged synchrony, whereas no significant synchrony was found during rest and the control task when played alone. While increased IBI synchrony was observed for La1 and La2, increased RSA synchrony was only found for La1. Further, positive affect and RSA synchrony were higher for negative than for positive lags at La1 indicating that changes in child affect/RSA preceded that of the mother. Positive affect was associated with higher IBI and RSA synchrony in the interactive tasks, although interestingly, it was more strongly related to the mother’s and child’s individual positive affect than to their shared positive affect. Further, relationships were only observed within but not across dyads.

The results support the hypothesis that mother–child interactions involve a coordination of both partner’s physiological states in response to each other’s emotional and behavioral cues. Such synchrony seems to occur primarily in the social interaction with others rather than when playing alone or watching a relaxing video together (see also^5^). However, significant IBI synchrony was found during both labyrinth tasks whereas RSA synchrony was only found in the first task (although RSA synchrony did not significantly differ between La1 and La2). IBI synchrony may increase during tasks that are challenging and emotionally charged^5^, which is likely the case for the labyrinth game. In contrast, RSA synchrony may be particularly responsible for a first physiological attunement between mother and child as a response to a stressor^33^. During the second block, such an attunement may no longer be necessary, or only to a lesser degree, as both partners are accustomed to the stressor (e.g., when a marble falls through the hole) and to their “roles in the game”. This however does not seem to be associated with a learning effect since the number of successes and failures (ball falling through the hole) did not significantly differ between La1 and La2. Alternatively, our data showed generally lower positive affect during the second compared to the first game, perhaps due to effects of fatigue or reduced joy of playing the game again. Thus, lower levels of positive affect might have resulted in lower physiological synchrony levels, although no correlation with positive affect was found across dyads. Further, differences between RSA and IBI in synchrony should be interpreted with caution since RSA and IBI have different signal properties, such as a relatively slow periodicity of RSA (for an example see Fig. 1), that could possibly affect synchrony calculations.

On average, concurrent and lagged synchrony values were positive and significantly higher than zero, indicating positive synchrony of IBI and RSA. This is in line with Lunkenheimer et al. who showed positive coregulation of RSA during three laboratory tasks (free play, clean up, puzzle task) in a community sample of mothers and their preschool children, but negative coregulation when externalizing behaviors were higher^7^. Since our analyses were based on group averages, some dyads may still show negative concordance; however, such differentiation with respect to certain risk factors is beyond the scope of the paper.

Further, results showed that significant IBI and RSA synchrony occurred both at positive and negative lags, although a stronger child leads/mother follows covariation was observed during La1 for positive affect and RSA synchrony. There are few studies which have examined bidirectional influences in physiological synchrony, however, based on different analytical methods (e.g., using a multilevel actor–partner interdependence model^34^). In contrast to our findings, there are two studies on preschoolers and adolescents which showed that parent HR/RSA activity positively predicted child HR/RSA activity but not vice versa^34,35^ and another study in kindergarten children which showed that during a problem-solving task, parent and child RSA activity reciprocally influenced each other in bidirectional patterns (parent RSA positively associated with child RSA and child RSA negatively associated with parent RSA) across 30-s epochs^6^. Here, our data indicate that mothers in a low-risk community sample were sensitive to the child’s affective cues. Thus, they may have perceived the child’s affective states and responded by matching their own affect and physiology. Future studies are needed to explore whether this is related to general measures of maternal sensitivity and how this is modulated by risk conditions, such as maternal depression. Finally, by increasing their own positive affect in response to their child’s, mothers may help children to up-regulate or sustain positive emotions. Parents emotion socialization behaviors, which includes how parents express and regulate their own emotions, as well as how they process and respond to their child’s, have been linked to youth positive emotions in intervention studies^16^. Thus, synchrony in positive affect could make the parent–child interaction more enjoyable, promote the development of attachment bonds and be a protective factor for the child’s socioemotional development. In line thereof, a recent meta-analysis showed that securely attached children experienced more global positive affect, less global negative affect and were better able to regulate emotions, pointing towards the relationship between positive emotions and parent–child attachment, albeit both directions of effects are likely^12^.

For our second research question, results showed that individual and shared positive affect were positively related to IBI and RSA synchrony within but not across dyads. These differential results suggest that changes in maternal and child IBI and RSA associated with changes in affect, i.e., presence or absence of positive facial affect, contributed to the synchronization of physiological activities in the labyrinth task (within-dyad regression analyses). However, more positive affect was not necessarily associated with higher physiological synchrony across-dyads (between-dyad correlation analyses). Further, in addition to the influence of emotional cues other unnoticed aspects of physical behavior or synchrony (e.g., physical proximity) might have impacted on physiological synchrony in this coordinated movement task.

Further, effects were larger (i.e., IBI and RSA synchrony were more strongly reduced) when we regressed out the mother’s and child’s individual positive affect compared to moments in which they shared positive affect. This suggests that positive affect is associated with a synchronization of physiological activities, although positive affect does not necessarily need to occur simultaneously. Synchrony may also arise in moments in which only one interaction partner shows positive affect, as would be the case for time-lagged effects. Further, perceiving positive affect of the interaction partner may elicit similar physiological responses, which may or may not be accompanied by expressions of affect. Besides perceiving and reacting to the partner’s emotions, in the current game, synchrony may arise as both partners are entrained to the task, e.g., showing similar physiological responses when the marble falls or nearly falls into the hole.

Taken together, these results suggest that physiological synchrony is more than an epiphenomenon of experiencing the same emotion simultaneously. Rather, it may be an ongoing transactional process between both partner’s physiology and observable behavior with bidirectional relationships between physiological and affect synchrony. It may arise in the interaction between parent and child when both experience and react to the other’s emotions as well as share the same emotions at the same time.

Of note is also that we found low stabilities, i.e., no significant correlations, between the first and second labyrinth game in IBI and RSA synchrony, while the amount of positive affect was positively correlated between the two games. On the one hand, this may point to problems with the reliability of physiological synchrony metrics, and certainly more research is needed to assess the reliability of various indices and analytical approaches. On the other hand, this raises the question of whether physiological synchrony should rather be understood as a state- or trait-level variable. Theoretically, caregiver-child interpersonal synchrony is thought to consolidate into relatively stable trajectories that may be related to the attachment relationship and shape the child’s development throughout life^36^. A moderate stability has been shown for behavioral measures (e.g., correlations between *r* = 0.25 and *r* = 0.51 for parent–child reciprocity between the first and fifth year of life^37^), yet, as far as we know, no study to date has examined the stability of physiological synchrony across tasks and time. In this regard, a low stability may indicate that mother–child physiological synchrony is more strongly related to the situation rather to relatively stable relationship characteristics, such as mother–child attachment.

A particular of strength of the study is that we combined rather novel approaches to analyse our data, i.e., estimating time-varying RSA^32^ and tracking the back-and-forth relationships between physiology and behavior during dyadic interactions^38^. This allowed us to examine parasympathetic activity with a finer temporal resolution as well as the relation of physiological synchrony and behavior on a second-by-second basis. Yet, these approaches are not strongly represented in current literature which makes our results more difficult to compare. Further, we focused on measures that represent parasympathetic activity or general ANS activity. Future studies may additionally consider isolated measures of the sympathetic nervous system (e.g., pre-ejection period), which may provide further information about coping with stressful environments and arousal during more challenging dyadic interactions^39^.

Several limitations should be noted. First, we investigated a rather unnatural mother-infant interaction, as both partners were not allowed to move freely or talk to each other, reducing the quantity and quality of interactive moments. Further, participants showed very few negative emotions during the tasks, making it impossible to discern the effects of positive vs. negative affect. Thus, future studies, may compare standardized laboratory tasks with more naturalistic tasks and contrast both positive and more stressful tasks, that require stronger emotion regulatory abilities. Here, we found no significant correlations between positive affect, coded on a second-by-second basis from the videos, and global post-task self-report ratings of emotional valence and arousal. Future studies may use moment-to-moment self-report measures of positive and negative affect to serve as an additional metric of emotional synchrony. Finally, here we decided to choose the same frequency bands for adult and child RSA synchrony analyses for consistency, while future studies may explore cross-frequency couplings. Keeping these caveats in mind, the current findings provide new insights into the fine-grade reciprocity of biobehavioral synchrony in low-risk parent–child dyads which need further validation in longitudinal studies.

## Methods

### Participants

A total of 48 children, aged between 8 and 10 years, and their mothers (mother–child dyads) were recruited through flyers in schools, paediatrician practices and youth centres, the social networks of the researchers and participants as well as advertisements in the intranet of the University Hospital RWTH Aachen. Participants were excluded if they had a history of any severe neurological, psychiatric or cardiological disorder. Data collection took place between 08/2019 and 08/2021. Due to the SARS-CoV2-pandemic during 2020 and 2021, the study was paused several times for a few months. Afterwards the study was continued under strict hygiene regulations (excluding subjects with any symptoms of a common cold, taking any medications that weakened the immune system (e.g., cortisone-based medication) or living together with a person in the household with an increased risk for a severe course of the Covid disease). A total of 19 dyads were tested before and 23 during the pandemic. Since no significant differences were found between dyads tested before and during the pandemic in IBI, RSA and affect synchrony, this variable was not further included as a covariate.

Of the initial sample, six dyads were excluded, four due to missing ECG data or issues with ECG data collection, one due to an experimental error and one due to an intake of an antidepressant of the mother. Thus, the final sample consisted of 42 mothers (*M*_*age*_ = 40.8 years, *SD* = 4.7 years) and their biological children (*M*_*age*_ = 9 years, *SD* = 1.2 years). Of these children, 23 (55%) were female. Median parental education was at university level and the majority of parents was legally married (81%).

Due to incorrect or missing heart beats, some dyads had missing data in one or more than one of the task conditions (for details see “ANS data acquisition and pre-processing” section). Additionally for one dyad, video data was missing. Thus, the actual sample size varied between *n* = 33 and *n* = 39 per condition (see Table 1).

Written informed consent was provided by the mothers for their own study participation as well as by both parents holding custody for their child’s participation. Children provided written informed assent. Participants were reimbursed for study participation. All methods were performed in accordance with the relevant guidelines and regulations and ethical approval was obtained by the Ethics Committee of the Medical Faculty, University Hospital RWTH Aachen (EK 076/19).

### Procedure and tasks

The current study was part of a larger investigation with two assessments approximately two to three weeks apart. In addition to the ANS recordings, the participant’s brain activities were measured using functional near-infrared spectroscopy (fNIRS). In the current article, only the first assessment and the ANS data are reported, the fNIRS data will be presented elsewhere.

Upon arrival in the lab, electrodes were attached to the participants’ bodies. This was done in the beginning to give the participants enough time to habituate to the procedure. Afterwards, the tasks were explained, and practice blocks provided. During the experiment, participants were seated next to each other in front of a table (for a picture see SI, Fig. S1). They were asked not to talk to each other and to rest their heads on chin rests in order to reduce movement artefacts (particularly important for fNIRS data quality). After each task, participants filled in Self-Assessment Manikin (SAM)^40^ ratings of their affect “How happy are you?” and arousal “How excited are you?”, each on a 9-point scale.

#### Rest

In the beginning of each experiment, mother and child watched a 5-min excerpt of a relaxing video on a computer screen, which was originally developed to obtain task-free fMRI “rest” scans in children. The video featured abstract shapes without a narrative or scene-cuts^41^. To prevent any communication and influences between participants, they were separated by a partition wall.

#### Joint labyrinth

After the rest block, dyads participated in a joint labyrinth task (e.g.^42^) which required them to jointly navigate a marble through a tilting labyrinth box, without falling in one of the holes (see SI, Fig. S2). Each player operated one dial, either tilting the labyrinth in the left–right or in the front-back direction, without touching the other’s dial. During the task, mother and child were asked not to talk to each other. Thus, the coordination of behavioral actions and responses mainly occurred at a nonverbal level (although participants laughed, sighed etc.). This task was played twice in blocks of 5 min each, referred to as ‘La1’ (first labyrinth task) and ‘La2’ (second labyrinth task). During each block, the mother–child interaction was recorded on video for later micro-coding analyses (see “Observational measures of the mother–child interaction” section). In addition, participants played two cooperative computer games (for details see^43^), which were presented alternatingly with La1 and La2 in a pseudo-randomized order, but were not considered in the current article.

#### Single labyrinth

At the end of the experiment, both mother and child played the labyrinth game individually for five minutes each. In the ‘Single’ task condition, each participant operated both dials. To ensure that participants did not influence or distract each other, they were again separated by a partition wall and did not play at the same time but one after another, while the other completed a different task (i.e., the mother filled out questionnaires and the child played an Etch-a-Sketch drawing game; data not analyzed). The child always completed the single labyrinth game before the mother to reduce wearing times of the fNIRS caps which were taken off directly after the game.

### ANS data acquisition and pre-processing

Electrocardiogram (ECG) was collected continuously using the 7-lead version of the VU-AMS5fs device (Vrije University, Amsterdam). A total of seven ECG leads were attached to the participants’ upper bodies using disposable pre-gelled Ag/AgCl spot electrodes (Kendall, former Covidien, Medtronics, USA) and after cleaning the skin with alcohol to ensure a low electrode resistance. Electrodes were placed slightly below the right collar bone, at the apex of the heart approximately at the level of the processus xiphoidius, between the lower two ribs of the right abdomen, at the top end of the sternum between both collar bones and on the xiphoid complex of the sternum. Two electrodes were attached at the back on the spine over the cervical vertebra C4 and between thoracic vertebrae T8 and T9^44^.

Cardiac data was imported into the VU-DAMS software (Version 4.3) for preprocessing. R-peaks were visually inspected and missing or incorrect peaks were corrected or removed. Since only dyads with no missing beats were considered in the synchrony analysis, this resulted in missing values: *n* = 8 missing in La1, *n* = 5 in La2, *n* = 9 in the Single and *n* = 3 in the Rest condition. Further, since RSA estimates cannot be derived for the first and final 15 seconds of the recording (see “RSA synchrony” section), all synchrony analyses were based on ~ 270s of data in each condition.

### IBI synchrony

IBI synchrony was calculated based on Reindl et al.^45^. First, IBI time series were resampled at 10 Hz as described in Berntson et al., deriving a time series of 1 s epochs with fixed on- and offsets to ensure temporal synchronization of adult’s and child’s epoch means time series^46^. For each epoch, the 10 samples were averaged into one IBI value per second. An epoch length of 1s was chosen to keep the analysis as consistent as possible to the RSA analysis (see “RSA synchrony” section). In order to remove linear and quadratic trends, a second order polynomial regression was applied to each epoch means time series^9^. The residuals of the polynomial regression were then subjected to Autoregressive Integrated Moving Average (ARIMA) modeling, with one autoregressive term, one moving average term, and integrated noise, and the resulting residuals were entered into the cross-correlation analysis^9,18,45^. Cross-correlations were calculated between the child’s and mother's residual time series for each condition separately and at lags between − 3 and + 3 s. At lag 0, the coefficients can be interpreted as the degree to which an IBI increase/decrease in one individual corresponds to an IBI increase/decrease in the other individual at the same time. Negative lags indicate that the child’s time series preceded the mother, i.e., an IBI increase/decrease of the child is followed by an IBI increase/decrease of the mother, while positive lags indicate that the mother’s time series preceded that of the child. Further, positive cross-correlation values describe changes in mother’s and child’s physiology in the same direction, while negative values indicate signal changes in opposite direction.

### RSA synchrony

RSA was estimated at 1s time intervals using the RSAseconds program^32^. First, the participant’s IBI series were interpolated at 4 Hz using a cubic spline, making the time series equidistant. Second, Peak Matched Multiple Windows (PM MW) tapering windows were applied, and short-time Fourier transform (STFT) was conducted on the time series with 32 s windows and 31 s overlap. To obtain power estimates, the squared sum of the STFT estimates was obtained in the 0.12–0.40 Hz frequency band, associated with respiration. Of note is that this frequency band has been recommended for adults^32^. In line with Oshri et al. (age range 9 to 12 years^47^) and to keep it consistent to the adult data, we used the same frequency band in children, although other studies use different frequency bands (e.g., Gentzler et al.: in 5- to 13-year-olds^30^; Woody et al. in 7- to 11-year-olds^20^). Heart rate and respiratory rate estimates in children are inconsistent amongst existing reference ranges but are already relatively close to values observed in adults^48^. Estimates at each epoch were then natural log transformed. Thus, for each participant and task, one RSA time series was derived, sampled at 1s epochs.

Next, RSA synchrony was calculated as described for IBI synchrony. Specifically, we first applied a second order polynomial regression to the RSA time series, then subjected the residuals to ARIMA modelling and finally, calculated the cross-correlation between the child’s and mother’s residual time series at lags – 3 s (child leads) to + 3 s (mother leads).

### Observational measures of the mother–child interaction

Mother–child interaction during the labyrinth task was micro-coded in ELAN Linguistic Annotator (5.9) (Max Planck Institute for Psycholinguistics, Nijmegen) by a trained observer along three scales (affect, partner-directed attention and body movements), adapted from Neale et al.^49^. Mother’s and child’s behaviour was coded separately on a second-by-second basis according to the presence or absence (1/0) of activity in each dimension. Specifically, affect was coded to be present if the participant showed negative emotions (1.1), e.g., frowning, sighing, or positive emotions (1.2), e.g., laughing, smiling. Since negative emotions occurred very rarely (*M* = 0.27%, *SD* = 0.45%), only positive emotions were considered in the subsequent analyses. Body movements were coded to be present (1) if the participant showed stronger movements of the upper body, e.g., turning around, lifting, swinging, fidgeting of the arms, except for operating the dial. Finally, attention was coded to be either directed towards the partner (1), for instance, by touching or looking at the partner, or on the game (0) (for details on the coding scheme see SI, Fig. S2). Since shared body movements and partner-directed attention occurred on average in less than 5% of the total task time and were restricted by the experimental design due to the chin rests, these were not further considered in the analyses. Further, both variables were unrelated to shared positive affect (correlation to shared body movements: *r* = 0.140, *p* = 0.38; correlation to shared partner-directed attention: *r* = 0.246, *p* = 0.25).

From the micro-coding, two *individual* time series were derived, one for the mother’s positive affect and one for the child’s positive affect (0 = neutral or negative affect, 1 = positive affect), both sampled on a one-second basis. In addition, to these *individual* time series of mother and child, a *dyadic* time series was calculated for shared positive affect (affect synchrony), whereby a score of 1 was assigned if both mother and child showed positive affect, while all other instances were coded with 0.

To calculate inter-rater reliability, a randomly selected subset of videos (30% of videos) was coded by a second observer and intraclass correlations were computed. Cronbach’s alpha was 0.89 for shared positive affect, 0.94 for child’s and 0.88 for mother’s individual positive affect.

### Statistical analysis

Statistical analyses were conducted in IBM SPSS Statistics 27 and R version 3.6.2 (R Core Team, 2019). Prior to the analyses, data were checked for outlying values and there were no values were higher/lower than 3 SD from the mean.

To examine how ANS and affect synchrony was influenced by the experimental condition (Research Question 1), we first compared IBI, RSA and affect synchrony of mother–child dyads to synchrony of shuffled adult–child pairs who were not part of the same dyad^50^. Specifically, we constructed sets of shuffled pairs for each child by permuting all adult partners (N_adult_ − 1) and for each adult by permuting all child partners (N_child_ − 1), while keeping the condition (Rest, Single, La1, La2) fixed. Finally, we derived a dyad- and condition-specific mean shuffled pair synchrony value by averaging across the synchrony values of the child’s and adult’s shuffled pair sets in the respective condition. This way all possible combinations of permuted pairs were considered in the analysis but by deriving a dyad- and condition-specific shuffled pair value this accounted for differences in dyads and conditions, e.g., in mean IBI and mean RSA, that may affect synchrony values.

Thus, for each mother–child dyad we obtained one actual and one mean shuffled pair IBI, RSA, affect synchrony value. These values were than compared to synchrony values of actual mother–child dyads using dependent sample *t*-tests. Next, we directly compared IBI/RSA synchrony between conditions using a series of linear mixed models with IBI/RSA synchrony at each of the lags as the dependent variable, condition (Rest, Single, La1, La2) as fixed effect and subject as a random intercept. Since affect synchrony was calculated only for La1 and La2 and not for Rest and Single, linear mixed models were only calculated for IBI and RSA synchrony. Finally, we compared IBI, RSA and affect synchrony at positive and negative lags using dependent sample *t*-tests. La1 and La2 were considered separately in the analyses since there were mostly non-significant correlations between La1 and La2 in IBI and RSA synchrony (for RSA synchrony correlations were found ranging from 0.38 to 0.42 for positive lags only).

To assess the relationship between ANS synchrony and positive affect within and across dyads (Research Question 2), we first calculated bivariate Pearson correlations between IBI and RSA synchrony values as well as mother’s and child’s individual and shared positive affect at lag 0 (across-dyad analyses). Next, we regressed out the mother’s and child’s individual affect as well as their shared affect from their IBI/RSA time series in two separate analyses to examine whether this significantly reduced ANS synchrony (within-dyad analyses; adapting the approach by Piazza et al.^38^). Such a reduced synchrony would indicate that individual or shared positive affect modulates ANS synchrony within dyads. To do so, we first performed linear regressions using the mother’s and child’s individual affect time series and second, using their shared positive affect time series to predict the mother’s and child’s ANS responses. We regressed out each affect time series at all possible lags from − 10 (ANS leading) to + 10 s (behavior leading) by shifting the two relative to each other in 1s increments. This analysis completely removed the influence of positive affect from the ANS signals, regardless of their temporal alignment. Regression analyses were performed for each participant separately, partialling out person-specific associations. Thereafter, we calculated ANS synchrony by computing the cross-correlation of the ‘cleaned’ ANS signals of mother and child at all lags (− 3 to + 3). Thus, for each lag and condition, we derived two IBI/RSA synchrony values, one with mother’s and child’s individual affect and one with their shared affect removed. For each condition and lag, we first compared ‘cleaned’ physiological synchrony values to ‘uncleaned’ values using dependent sample *t*-tests. To control for the possibility that synchrony was reduced, e.g., because the regression residuals have a lower variability, significant results were validated again by comparing actual and shuffled synchrony values using dependent sample *t*-tests. Sets of shuffled pairs were created for each participant and condition by regressing out the affect time series of all other children from the child’s ANS responses and by regressing out the affect time series of all other mothers from the mother’s ANS responses while keeping the condition (La1, La2) fixed. In a further step, we compared the effects of regressing out mother’s and child’s individual affect to the effects of regressing out their shared affect. For each analysis, a lower synchrony value indicated a significant modulation of physiological synchrony, i.e., when participant’s positive affect is partialled out, physiological synchrony is reduced.

Linear mixed models were conducted in the R package ‘lme4’^51^. Final models were fitted by REML and *p*-values were obtained using the summary function of the R package ‘lmerTest’^52^ with the Satterthwaite approximation for the degrees of freedom. For significant condition effects, pairwise contrasts were calculated using the R package ‘emmeans’^53^. All *p*-values were corrected using false-discovery rate correction^54^. Specifically, *p* values were corrected across lags per condition and synchrony measure (IBI, RSA, affect) for the *t*-tests, across lags and per synchrony measure for the linear mixed models as well as across the number of pairwise comparisons per linear mixed model. Both corrected and uncorrected *p* values are reported in the supplement. Effect sizes for linear mixed models were calculated using the R package ‘r2glmm’^55^ and the approach by Nakagawa and Schielzeth^56^.

### Supplementary Information

Supplementary Information.

## Data Availability

The datasets generated and analysed during the current study are available from the corresponding authors on reasonable request.
