# Supplementary material for: Concurrent and lagged physiological synchrony during mother–child interaction and their relationship to positive affect in 8- to 10-year-old children
Source: Sci Rep. 2023 Oct 18;13:17744. doi: 10.1038/s41598-023-43847-8 (PMC10584844; doi:10.1038/s41598-023-43847-8)
Supplement: Supplementary file 1 — Supplementary Information. [file 41598_2023_43847_MOESM1_ESM.pdf]

Supplementary Information

Concurrent and lagged physiological synchrony during mother-child interaction and their relationship to positive affect in 8- to 10-year-old children

Yasemin Capraz [1], Kerstin Konrad [1,2] & Vanessa Reindl [1,2,3*]

[1] Child Neuropsychology Section, Department of Child and Adolescent Psychiatry, Psychosomatics and Psychotherapy, Medical Faculty, RWTH Aachen University, Germany

[2] JARA-Brain Institute II, Molecular Neuroscience and Neuroimaging, RWTH Aachen & Research Centre Juelich, Germany

[3] Psychology, School of Social Sciences, Nanyang Technological University, Singapore S639818, Republic of Singapore

Table S1. Means, standard deviations and sample range for IBI synchrony, RSA synchrony, shared and individual positive affect at lag 0.

**Variable**		***n***	***M***	***SD***	***Min***	***Max***
**IBI Synchrony**	**1. La1**	34	.095	.071	.002	.251
	**2. La2**	37	.099	.061	-.035	.215
	**3. Single**	33	.002	.073	-.160	.133
	**4. Rest**	39	.017	.052	-.084	.137
**RSA Synchrony**	**5. La1**	34	.084	.171	-.286	.398
	**6. La2**	37	.094	.187	-.350	.428
	**7. Single**	33	-.037	.146	-.364	.257
	**8. Rest**	39	.023	.180	-.268	.330
**Shared pos. Affect**	**9. La1**	41	.071	.067	.000	.256
	**10. La2**	41	.041	.044	.000	.178
**Mother pos. affect**	**11. La1**	41	.135	.103	.000	.448
	**12. La2**	41	.090	.074	.000	.311
**Child pos. affect**	**13. La1**	41	.122	.095	.000	.404
	**14. La2**	41	.079	.073	.000	.267

Note. La1 = first joint labyrinth game, La2 = second joint labyrinth game, Single = single labyrinth game, Rest = rest video.

Table S2a. Results of a paired sample t-test comparing the number of times the ball fell into the hole (failure) for the joint labyrinth game**.**

	**La1 [Mean (SD)]**	**La2 [Mean (SD)]**	**t-test [*t*(df), p]**
**Failure**	13.31 (3.70)	14.13 (3.29)	-1.09 (38), .283

La1 = first joint labyrinth game, La2 = second joint labyrinth game.

Table S2b. Results of a Wilcoxon signed rank test comparing the number of successful rounds (Success) for the joint labyrinth game**.**

	**La1 [Mean (SD)]**	**La2 [Mean (SD)]**	**Wilcoxon test [z, p]**
**Success**	1.08 (1.69)	1.41 (1.60)	1.85, .065

La1 = first joint labyrinth game, La2 = second joint labyrinth game.

Table S3a. Results of paired sample t-tests comparing synchrony of actual and shuffled pairs for the first (La1) and second (La2) joint labyrinth game.

		**IBI synchrony**			**RSA synchrony**		
	**Lag**	**Actual [Mean (SD)]**	**Shuffled [Mean (SD)]**	**t-test [t(df), p (p_{adj})]**	**Actual [Mean (SD)]**	**Shuffled [Mean (SD)]**	**t-test [t(df), p (p_{adj})]**
**La1**	**-3**	.0213 (.0632)	.0055 (.0085)	$t(33) = -1.48$, $p = .149$ (.174)	.1052 (.1703)	.0026 (.0212)	$t(33) = -3.48$, $p = .001$ (.002)
	**-2**	.0639 (.0714)	.0006 (.0085)	$t(33) = -5.09$, $p < .001$ (.002)	.1056 (.1656)	.0014 (.0200)	$t(33) = -3.67$, $p = .001$ (.002)
	**-1**	.0869 (.0681)	.0007 (.0083)	$t(33) = -7.32$, $p < .001$ (.002)	.0991 (.1614)	-.0008 (.0180)	$t(33)= -3.60$, $p = .001$ (.002)
	**0**	.0930 (.0700)	.0029 (.0085)	$t(33) = -7.49$, $p < .001$ (.002)	.0818 (.1605)	-.0027 (.0208)	$t(33) = -3.07$, $p = .004$ (.007)
	**+1**	.0729 (.0590)	.0035 (.0073)	$t(33) = -6.77$, $p < .001$ (.002)	.0613 (.1617)	-.0044 (.0228)	$t(33) = -2.35$, $p = .025$ (.035)
	**+2**	.0228 (.0751)	.0028 (.0078)	$t(33) = -1.54$, $p = .132$ (.174)	.0356 (.1600)	-.0051 (.0241)	t(33)= -1.47, $p = .152$ (.177)
	**+3**	.0124 (.0685)	.0030 (.0075)	$t(33) = -.81$, $p = .423$ (.423)	.0130 (.1538)	-.0054 (.0245)	$t(33) = -.68$, $p = .500$ (.500)
**La2**	**-3**	-.0154 (.0696)	.0010 (.0065)	$t(36) = 1.42$, $p = .164$ (.196)	.0206 (.1632)	.0037 (.0152)	$t(36) = -.61$, $p = .543$ (.559)
	**-2**	.0206 (.0863)	.0006 (.0077)	$t(36) = -1.41$, $p = .168$ (.196)	.0373 (.1656)	.0071 (.0026)	$t(36) = -1.07$, $p = .290$ (.461)
	**-1**	.0654 (.0737)	-.0010 (.0012)	t(36) = -5.45, $p < .001$ (.002)	.0502 (.1796)	.0091 (.0164)	$t(36) = -1.36$, $p = .183$ (.461)
	**0**	.0980 (.0583)	.0005 (.0076)	$t(36) = -9.85$, $p < .001$ (.002)	.0554 (.1879)	.0104 (.0164)	$t(36) = -1.43$, $p = .162$ (.461)
	**+1**	.0670 (.0781)	.0004 (.0053)	$t(36) = -5.23$, $p < .001$ (.002)	.0514 (.1940)	.0097 (.0158)	$t(36) = -1.29$, $p = .206$ (.461)
	**+2**	.0262 (.0671)	-.0004 (.0084)	$t(36) = -2.45$, $p = .019$ (.033)	.0394 (.1889)	.0083 (.0151)	$t(36) = -.99$, $p = .329$ (.461)
	**+3**	.0142 (.0801)	.0055 (.0078)	$t(36) = -.65$, $p = .518$ (.518)	.0229 (.1795)	.0053 (.0147)	$t(36) = -.59$, $p= .559$ (.559)

Note. adj = *FDR* adjusted *p*-values. Lags are measured in seconds.
La1 = first joint labyrinth game, La2 = second joint labyrinth game.

Table S3b. Results of paired sample t-tests comparing synchrony of actual and shuffled pairs for the single labyrinth game (Single) and Rest.

		**IBI synchrony**			**RSA synchrony**		
	**Lag**	**Actual [Mean (SD)]**	**Shuffled [Mean (SD)]**	**t-test [*t*(df), *p* (*p* $_{adj}$)]**	**Actual [Mean (SD)]**	**Shuffled [Mean (SD)]**	**t-test [*t*(df), *p* (*p* $_{adj}$)]**
**Single**	**-3**	.0033 (.0702)	.0025 (.0073)	*t*(32) = -.06, *p* = .950 (.973)	-.0521 (.1530)	-.0021 (.0211)	*t*(32) = 1.99, *p* = .055 (.182)
	**-2**	.0022 (.0690)	.0018 (.0066)	*t*(32) = -.03, *p*= .973 (.973)	-.0519 (.1446)	-.0038 (.0217)	*t*(32) = 2.03, *p* = .051 (.182)
	**-1**	-.0184 (.0564)	.0030 (.0091)	*t*(32) = 2.21, *p* = .034 (.238)	-.0475 (.1428)	-.0048 (.0224)	*t*(32) = 1.82, *p* = .078 (.182)
	**0**	.0051 (.0714)	-.0012 (.0079)	t(32) = -.50, *p* = .623 (.973)	-.0400 (.1436)	-.0056 (.0227)	t(32) = 1.45, *p* = .156 (.273)
	**+1**	-.0041 (.0684)	-.0004 (.0071)	*t*(32) = .31, *p* = .756 (.973)	-.0327 (.1535)	-.0056 (.0225)	*t*(32) = 1.06, *p* = .296 (.414)
	**+2**	.0061 (.0554)	.0007 (.0074)	*t*(32) = -.54, *p* = .592 (.973)	-.0260 (.0281)	-.0051 (.0038)	*t*(32) = .77, *p* = .447 (.522)
	**+3**	.0081 (.0621)	.0001 (.0064)	t(32) = -.75, *p* = .458 (.973)	-.0227 (.1702)	-.0039 (.0214)	*t*(32) = .65, *p* = .523 (.523)
**Rest**	**-3**	.0070 (.0600)	.0016 (.0072)	*t*(38) = -.55, *p* = .587 (.996)	-.0049 (.1673)	-.0006 (.0138)	*t*(38) = .16, *p* = .874 (.874)
	**-2**	.0056 (.0581)	.0038 (.0095)	*t*(38) = -.19, *p* = .848 (.996)	.0036 (.1712)	-.0008 (.0134)	*t*(38) = -.16, *p* = .874 (.874)
	**-1**	.0041 (.0587)	.0042 (.0075)	t(38) = .01, *p* = .996 (.996)	.0108 (.1688)	-.0005 (.0133)	*t*(38) = -.42, *p* = .677 (.874)
	**0**	.0142 (.0560)	.0018 (.0086)	*t*(38) = -1.39, *p* = .172 (.917)	.0211 (.1677)	-.0005 (.0143)	*t*(38) = -.80, *p* = .427 (.859)
	**+1**	.0058 (.0596)	.0049 (.0091)	*t*(38) = -.09, *p* = .925 (.996)	.0243 (.1651)	-.0000 (.0154)	*t*(38) = -.92, *p* = .363 (.859)
	**+2**	-.0016 (.0678)	-.0002 (.0086)	t(38) = .13, *p* = .896 (.996)	.0267 (.1639)	.0006 (.0167)	*t*(38) = -.99, *p* = .328 (.859)
	**+3**	.0081 (.0477)	-.0009 (.0091)	*t*(38) = -1.14, *p* = .262 (.917)	.0196 (.1604)	.0017 (.0177)	*t*(38) = -.70, *p* = .491 (.859)

Note. adj = *FDR* adjusted *p*-values. Lags are measured in seconds.
Single = single labyrinth game, Rest = rest video.

Table S4. Main effects of task in linear mixed models predicting IBI / RSA synchrony.

	IBI synchrony					RSA synchrony				
**Lags**	**Sum Sq**	**Mean Sq**	**df**	**F**	***p* (*p* adj)**	**Sum Sq**	**Mean Sq**	**df**	**F**	***p* (*p* adj)**
**-3**	.024	.008	3	1.90	.132 (.185)	.440	.147	3	5.40	.002 (.007)
**-2**	.083	.028	3	5.41	.001 (.002)	.438	.146	3	5.43	.001 (.007)
**-1**	.261	.087	3	20.86	<.001 (.002)	.388	.129	3	4.70	.004 (.009)
**0**	.273	.091	3	22.96	<.001 (.002)	.277	.092	3	3.35	.022 (.039)
**1**	.178	.059	3	13.29	<.001 (.002)	.178	.059	3	2.12	.101 (.141)
**2**	.019	.006	3	1.44	.234 (.273)	.098	.033	3	1.18	.321 (.375)
**3**	.001	.000	3	.06	.979 (.979)	.054	.018	3	.67	.573 (.573)

Note. adj = *FDR* adjusted *p*-values. Lags are measured in seconds.

Table S5a. Post hoc analyses comparing synchrony in the first joint labyrinth game (La1) to synchrony in the single labyrinth game (Single).

	IBI synchrony			RSA synchrony		
**Lags**	**La1 [Mean (SD)]**	**Single [Mean (SD)]**	**t-test [t(df), p_{adj}]**	**La1 [Mean (SD)]**	**Single [Mean (SD)]**	**t-test [t(df), p_{adj}]**
**-3**				.105 (.028)	-.052 (.029)	$t(111) = 3.898$, $p = .001$
**-2**	.064 (.012)	.002 (.013)	$t(110) = 3.517$, $p = .002$	.106 (.028)	-.052 (.029)	$t(111) = 3.924$, $p = .001$
**-1**	.087 (.011)	-.018 (.011)	$t(110) = 6.653$, $p < .001$	.099 (.029)	-.048 (.029)	$t(111) = 3.606$, $p = .003$
**0**	.093 (.011)	.005 (.011)	$t(109) = 5.702$, $p < .001$	.081 (.029)	-.040 (.029)	$t(111) = 2.982$, $p = .021$
**1**	.073 (.012)	-.004 (.012)	$t(110) = 4.719$, $p < .001$			

Note. adj = *FDR* adjusted *p*-values. are measured in seconds.
La1 = first joint labyrinth game, Single = single labyrinth game.

Table S5b. Post hoc analyses comparing synchrony in the first joint labyrinth game (La1) to synchrony during Rest.

	IBI synchrony			RSA synchrony		
**Lags**	**La1 [Mean (SD)]**	**Rest [Mean (SD)]**	**t-test [t(df), p_{adj}]**	**La1 [Mean (SD)]**	**Rest [Mean (SD)]**	**t-test [t(df), p_{adj}]**
**-3**				.105 (.028)	-.003 (.026)	$t(107) = 2.839$, $p = .016$
**-2**	.064 (.012)	.006 (.011)	$t(107) = 3.479$, $p = .002$	.106 (.028)	.005 (.026)	$t(107) = 2.637$, $p = .029$
**-1**	.087 (.011)	.003 (.010)	$t(107) = 5.574$, $p < .001$	.099 (.029)	.013 (.026)	$t(107) = 2.241$, $p = .054$
**0**	.093 (.011)	.011 (.010)	$t(106) = 5.563$, $p < .001$	.081 (.029)	.024 (.026)	$t(107) = 1.486$, $p = .210$
**1**	.073 (.012)	.003 (.011)	$t(107) = 4.467$, $p < .001$			

Note. adj = *FDR* adjusted *p*-values. Lags are measured in seconds.
La1 = first joint labyrinth game, Rest = rest video.

Table S5c. Post hoc analyses comparing synchrony in the second joint labyrinth game (La2) to synchrony in the single labyrinth game (Single).

	IBI synchrony			RSA synchrony		
**Lags**	**La2 [Mean (SD)]**	**Single [Mean (SD)]**	**t-test [t(df), p_{adj}]**	**La2 [Mean (SD)]**	**Single [Mean (SD)]**	**t-test [t(df), p_{adj}]**
**-3**				.021 (.027)	-.052 (.029)	$t(107) = 1.841$, $p = .103$
**-2**	.021 (.012)	.002 (.013)	$t(106) = 1.071$, $p = .430$	.037 (.027)	-.052 (.029)	$t(107) = 2.270$, $p = .050$
**-1**	.065 (.011)	-.018 (.011)	$t(106) = 5.412$, $p < .001$	.050 (.027)	-.048 (.029)	$t(107) = 2.455$, $p = .047$
**0**	.097 (.011)	.005 (.011)	$t(105) = 6.124$, $p < .001$	.056 (.028)	-.040 (.029)	$t(106) = 2.408$, $p = .053$
**1**	.067 (.011)	-.004 (.012)	$t(106) = 4.449$, $p < .001$			

Note. adj = *FDR* adjusted *p*-values. Lags are measured in seconds.
La2 = second joint labyrinth game, Single = single labyrinth game.

Table S5d. Post hoc analyses comparing synchrony in the second joint labyrinth game (La2) to synchrony during Rest.

	IBI synchrony			RSA synchrony		
**Lags**	**La2 [Mean (SD)]**	**Rest [Mean (SD)]**	**t-test [t(df), p_{adj}]**	**La2 [Mean (SD)]**	**Rest [Mean (SD)]**	**t-test [t(df), p_{adj}]**
**-3**				.021 (.027)	-.003 (.026)	$t(108) = 0.643$, $p = .521$
**-2**	.021 (.012)	.006 (.011)	$t(108) = 0.912$, $p = .437$	.037 (.027)	.005 (.026)	$t(108) = 0.862$, $p = .391$
**-1**	.065 (.011)	.003 (.010)	$t(108) = 4.248$, $p < .001$	.050 (.027)	.013 (.026)	$t(108) = 0.995$, $p = .322$
**0**	.097 (.011)	.011 (.010)	$t(107) = 5.990$, $p < .001$	.056 (.028)	.024 (.026)	$t(108) = 0.844$, $p = .481$
**1**	.067 (.011)	.003 (.011)	$t(108) = 4.177$, $p < .001$			

Note. adj = *FDR* adjusted *p*-values. Lags are measured in seconds.
La2 = second joint labyrinth game, Rest = rest video.

Table S5e. Post hoc analyses comparing synchrony in the first joint labyrinth game (La1) to synchrony in the second joint labyrinth game (La2).

	**IBI synchrony**			**RSA synchrony**		
**Lags**	**La1 [Mean (SD)]**	**La2 [Mean (SD)]**	**t-test [*t*(df), *p* $_{adj}$]**	**La2 [Mean (SD)]**	**Rest [Mean (SD)]**	**t-test [*t*(df), *p* $_{adj}$]**
**-3**				.105 (.028)	.021 (.027)	t(111) = 2.156, *p* = .067
**-2**	.064 (.012)	.021 (.012)	t(110) = 2.539, *p* = .025	.106 (.028)	.037 (.027)	t(111) = 1.751, *p* = .124
**-1**	.087 (.011)	.065 (.011)	t(110) = 1.394, *p* = .168	.099 (.029)	.050 (.027)	t(111) = 1.238, *p* = .262
**0**	.093 (.011)	.097 (.011)	t(109) = -.290, *p* = .773	.081 (.029)	.056 (.028)	t(110) = .645, *p* = .520
**1**	.073 (.012)	.067 (.011)	t(110) = .376, *p* = .708			

Note. adj = *FDR* adjusted *p*-values. Lags are measured in seconds.
La1 = first joint labyrinth game, La2 = second joint labyrinth game.

Table S6. Results of paired sample t-tests comparing synchrony of actual and shuffled pairs for **shared positive affect.**

		**shared positive affect**		
	**Lag**	**Actual [Mean (SD)]**	**Shuffled [Mean (SD)]**	**t-test [*t*(df), p (p $_{adj}$)]**
**La1**	**-3**	.044 (.047)	.016 (.011)	$t(40) = -5.031$, $p = .001$ (.001)
	**-2**	.052 (.059)	.016 (.011)	$t(40) = -4.749$, $p < .000$ (.001)
	**-1**	.065 (.065)	.016 (.012)	$t(40) = -5.754$, $p < .001$ (.001)
	**0**	.071 (.067)	.016 (.011)	$t(40) = -6.229$ $p < .001$ (.001)
	**+1**	.062 (.059)	.016 (.011)	$t(40) = -6.137$, $p < .001$ (.001)
	**+2**	.044 (.047)	.016 (.012)	$t(40) = -5.073$, $p < .001$ (.001)
	**+3**	.029 (.037)	.016 (.012)	$t(40) = -3.126$, $p = .003$ (.003)
**La2**	**-3**	.025 (.029)	.007 (.006)	$t(40) = -4.836$, $p < .001$ (.001)
	**-2**	.030 (.037)	.007 (.006)	$t(40) = -4.570$, $p < .001$ (.001)
	**-1**	.037 (.042)	.007 (.006)	$t(40) = -5.241$, $p < .001$ (.001)
	**0**	.041 (.044)	.007 (.006)	$t(40) = -5.489$, $p < .001$ (.001)
	**+1**	.035 (.038)	.007 (.006)	$t(40) = -5.460$, $p < .001$ (.001)
	**+2**	.025 (.029)	.007 (.006)	$t(40) = -4.804$, $p < .001$ (.001)
	**+3**	.016 (.022)	.007 (.006)	$t(40) = -3.126$, $p = .001$ (.001)

Note. adj = *FDR* adjusted *p*-values. Lags are measured in seconds.
La1 = first joint labyrinth game, La2 = second joint labyrinth game.

Table S7. Results of paired sample t-tests comparing synchrony of actual dyads **before and after regression of individual positive affect**.

		**IBI synchrony**			**RSA synchrony**		
	**Lag**	**Before [Mean (SD)]**	**After [Mean (SD)]**	**t-test [*t*(df), *p* (*p* adj)]**	**Before [Mean (SD)]**	**After [Mean (SD)]**	**t-test [*t*(df), *p* (*p* adj)]**
**La1**	**-3**	.0235 (.0629)	.0145 (.0541)	t(32) = 1.419, *p* = .166 (.194)	.1178 (.1560)	.0822 (.1651)	t(32) = 2.769, *p* = .009 (.011)
	**-2**	.0677 (.0687)	.0328 (.0752)	t(32) = 4.714, *p* = .000 (.000)	.1188 (.1491)	.0779 (.1622)	t(32) = 3.244, *p* = .003 (.005)
	**-1**	.0878 (.0690)	.0437 (.0699)	t(32) = 5.145, *p* = .000 (.000)	.1118 (.1458)	.0663 (.1671)	t(32) = 3.373, *p* = .002 (.005)
	**0**	.0941 (.0708)	.0569 (.0739)	t(32) = 4.528, *p* = .000 (.000)	.0930 (.1490)	.0437 (.1751)	t(32) = 3.263, *p* = .003 (.005)
	**+1**	.0710 (.0587)	.0415 (.0540)	t(32) = 5.305, *p* = .000 (.000)	.0704 (.1550)	.0198 (.1804)	t(32) = 3.185, *p* = .003 (.005)
	**+2**	.0219 (.0760)	.0004 (.0702)	t(32) = 4.287, *p* = .000 (.000)	.0424 (.1574)	-.0060 (.1789)	t(32) = 3.042, *p* = .005 (.007)
	**+3**	.0128 (.0695)	.0076 (.0651)	t(32) = .977, *p* = .336 (.336)	.0178 (.1536)	-.0227 (.1714)	t(32) = 2.614, *p* = .014 (.014)
**La2**	**-3**	-.0162 (.0704)	-.0255 (.0689)	t(35) = 1.489, *p* = .145 (.145)	.0160 (.1630)	-.0058 (.1533)	t(35) = 1.780, *p* = .084 (.084)
	**-2**	.0189 (.0869)	.0035 (.0926)	t(35) = 2.238, *p* = .032 (.045)	.0334 (.1662)	.0050 (.1541)	t(35) = 2.255, *p* = .031 (.036)
	**-1**	.0685 (.0723)	.0428 (.0671)	t(35) = 4.344, *p* = .000 (.000)	.0474 (.1813)	.0129 (.1697)	t(35) = 2.572, *p* = .015 (.021)
	**0**	.0948 (.0557)	.0634 (.0697)	t(35) = 4.253, *p* = .000 (.000)	.0537 (.1902)	.0119 (.1799)	t(35) = 2.817, *p* = .008 (.014)
	**+1**	.0685 (.0786)	.0372 (.0817)	t(35) = 5.009, *p* = .000 (.000)	.0509 (.1967)	.0032 (.1888)	t(35) = 2.930, *p* = .006 (.014)
	**+2**	.0290 (.0659)	.0067 (.0670)	t(35) = 3.428, *p* = .002 (.004)	.0398 (.1915)	-.0128 (.1870)	t(35) = 2.999, *p* = .005 (.014)
	**+3**	.0151 (.0810)	.0036 (.0746)	t(35) = 2.055, *p* = .047 (.055)	.0241 (.1819)	-.0285 (.1851)	t(35) = 2.960, *p* = .005 (.014)

Note. adj = *FDR* adjusted *p*-values. Lags are measured in seconds.
La1 = first joint labyrinth game, La2 = second joint labyrinth game.

Table S8. Results of paired sample t-tests comparing synchrony of actual and shuffled pairs after regression of mother's and child's **individual positive affect**

		**IBI synchrony**			**RSA synchrony**		
	**Lag**	**Actual [Mean (SD)]**	**Shuffled [Mean (SD)]**	**t-test [t(df), p (p $_{adj}$)]**	**Actual [Mean (SD)]**	**Shuffled [Mean (SD)]**	**t-test [t(df), p (p $_{adj}$)]**
**La1**	**-3**	.015 (.054)	.020 (.057)	t(32) = 1.061, p = .297 (.347)	.082 (.165)	.103 (.150)	t(32) = 1.428, p = .163 (.163)
	**-2**	.033 (.075)	.060 (.067)	t(32) = 3.990, p < .001 (.001)	.078 (.162)	.103 (.144)	t(32) = 1.824, p = .078 (.091)
	**-1**	.044 (.070)	.076 (.071)	t(32) = 4.463, p < .001 (.001)	.066 (.167)	.096 (.142)	t(32) = 2.122, p = .042 (.069)
	**0**	.057 (.074)	.084 (.069)	t(32) = 3.601, p = .001 (.001)	.044 (.176)	.076 (.144)	t(32) = 2.277, p = .030 (.069)
	**+1**	.042 (.054)	.063 (.056)	t(32) = 4.556, p < .001 (.001)	.020 (.180)	.053 (.147)	t(32) = 2.335, p = .026 (.069)
	**+2**	.000 (.070)	.015 (.073)	t(32) = 3.741, p = .001 (.001)	-.006 (.179)	.025 (.149)	t(32) = 2.278, p = .030 (.069)
	**+3**	.008 (.065)	.010 (.066)	t(32) = .447, p = .658 (.658)	-.023 (.171)	.005 (.146)	t(32) = 2.044, p = .049 (.069)
**La2**	**-3**	-.025 (.069)	-.020 (.068)	t(35) = 1.062, p = .295 (.295)	-.006 (.153)	.007 (.149)	t(35) = 1.188, p = .243 (.243)
	**-2**	.004 (.093)	.016 (.085)	t(35) = 2.044, p = .049 (.057)	.005 (.154)	.024 (.151)	t(35) = 1.654, p = .107 (.125)
	**-1**	.043 (.067)	.061 (.071)	t(35) = 3.453, p = .001 (.002)	.013 (.170)	.038 (.166)	t(35) = 2.065, p = .046 (.064)
	**0**	.063 (.070)	.088 (.058)	t(35) = 4.330, p < .001 (.002)	.012 (.180)	.044 (.175)	t(35) = 2.397, p = .022 (.039)
	**+1**	.037 (.082)	.061 (.076)	t(35) = 4.005, p < .001 (.002)	.003 (.189)	.042 (.181)	t(35) = 2.592, p = .014 (.033)
	**+2**	.007 (.067)	.020 (.062)	t(35) = 2.170, p = .037 (.052)	-.013 (.187)	.031 (.176)	t(35) = 2.722, p = .010 (.033)
	**+3**	.004 (.075)	.014 (.079)	t(35) = 2.354, p = .024 (.042)	-.028 (.185)	.017 (.168)	t(35) = 2.746, p = .009 (.033)

Note. adj = *FDR* adjusted *p*-values. Lags are measured in seconds.
La1 = first joint labyrinth game, La2 = second joint labyrinth game.

Table S9. Results of paired sample t-tests comparing synchrony of actual dyads **before and after regression of shared positive affect**.

		**IBI synchrony**			**RSA synchrony**		
	**Lag**	**Before [Mean (SD)]**	**After [Mean (SD)]**	**t-test [*t*(df), *p* (*p* adj)]**	**Before [Mean (SD)]**	**After [Mean (SD)]**	**t-test [*t*(df), *p* (*p* adj)]**
**La1**	**-3**	.0235 (.0629)	.0178 (.0565)	t(32) = 1.149, *p* = .259 (.302)	.1178 (.1560)	.1028 (.1739)	t(32) = 1.209, *p* = .236 (.236)
	**-2**	.0677 (.0687)	.0443 (.0765)	t(32) = 3.423, *p* = .002 (.014)	.1188 (.1491)	.1040 (.1668)	t(32) = 1.283, *p* = .209 (.236)
	**-1**	.0878 (.0690)	.0673 (.0769)	t(32) = 2.381, *p* = .023 (.034)	.1118 (.1458)	.0968 (.1642)	t(32) = 1.358, *p* = .184 (.236)
	**0**	.0941 (.0708)	.0761 (.0688)	t(32) = 2.363, *p* = .024 (.034)	.0930 (.1490)	.0756 (.1661)	t(32) = 1.541, *p* =.133 (.233)
	**+1**	.0710 (.0587)	.0540 (.0566)	t(32) = 2.939, *p* = .006 (.021)	.0704 (.1550)	.0492 (.1639)	t(32) = 1.921, *p* = .064 (.149)
	**+2**	.0219 (.0760)	.0088 (.0719)	t(32) = 2.557, *p* = .015(.034)	.0424 (.1574)	.0172 (.1603)	t(32) = 2.311, *p* = .027 (.130)
	**+3**	.0128 (.0695)	.0102 (.0680)	t(32) = .649, *p* = .521 (.521)	.0178 (.1536)	-.0055 (.1538)	t(32) = 2.173, *p* = .037 (.130)
**La2**	**-3**	-.0162 (.0704)	-.0229 (.0734)	t(35) = 1.705, *p* = .097 (.113)	.0160 (.1630)	.0108 (.1746)	t(35) = .572, *p* = .571 (.583)
	**-2**	.0189 (.0869)	.0139 (.0908)	t(35) = 1.283, *p* = .208 (.054)	.0334 (.1662)	.0282 (.1767)	t(35) = .581, *p* = .565 (.583)
	**-1**	.0685 (.0723)	.0593 (.0682)	t(35) = 2.088, *p* = .044 (.062)	.0474 (.1813)	.0425 (.1916)	t(35) = .554, *p* = .583 (.583)
	**0**	.0948 (.0557)	.0823 (.0650)	t(35) = 2.386, *p* = .023 (.054)	.0537 (.1902)	.0472 (.1964)	t(35) = .762, *p* = .451 (.583)
	**+1**	.0685 (.0786)	.0540 (.0750)	t(35) = 3.213, *p* = .003 (.021)	.0509 (.1967)	.0412 (.1971)	t(35) = 1.164, *p* = .252 (.583)
	**+2**	.0290 (.0659)	.0184 (.0653)	t(35) = 2.248, *p* = .031 (.054)	.0398 (.1915)	.0254 (.1830)	t(35) = 1.693, *p* = .099 (.347)
	**+3**	.0151 (.0810)	.0120 (.0809)	t(35) = .578, *p* = .567 (.567)	.0241 (.1819)	.0067 (.1712)	t(35) = 1.899, *p* = .066 (.347)

Note. adj = *FDR* adjusted *p*-values. Lags are measured in seconds.
La1 = first joint labyrinth game, La2 = second joint labyrinth game.

Table S10. Results of paired sample t-tests comparing synchrony of actual and shuffled pairs after regression of mother's and child's **shared positive affect**.

		**IBI synchrony**			**RSA synchrony**		
	**Lag**	**Actual [Mean (SD)]**	**Shuffled [Mean (SD)]**	**t-test [*t*(df), p (p_{adj})]**	**Actual [Mean (SD)]**	**Shuffled [Mean (SD)]**	**t-test [*t*(df), *p* (p_{adj})]**
**La1**	**-3**	.018 (.057)	.023 (.059)	*t*(32) = 1.301, *p* = .202 (.269)	.103 (.174)	.116 (.165)	*t*(32) = 1.246, *p* = .222 (.254)
	**-2**	.044 (.077)	.066 (.069)	*t*(32) = 3.536, *p* = .001 (.008)	.104 (.167)	.118 (.160)	t(32) = 1.513, *p* = .140 (.187)
	**-1**	.067 (.077)	.084 (.073)	*t*(32) = 2.276, *p* = .030 (.048)	.097 (.164)	.110 (.159)	*t*(32) = 1.725, *p* = .094 (.150)
	**0**	.076 (.069)	.092 (.071)	*t*(32) = 2.291, *p* = .029 (.048)	.076 (.166)	.090 (.162)	*t*(32) = 1.884, *p* = .069 (.150)
	**+1**	.054 (.057)	.069 (.059)	*t*(32) = 3.177, *p* = .003 (.012)	.049 (.164)	.064 (.164)	*t*(32) = 1.992, *p* = .055 (.150)
	**+2**	.009 (.072)	.019 (.076)	*t*(32) = 2.542, *p* = .016 (.043)	.017 (.160)	.034 (.166)	*t*(32) = 2.073, *p* = .046 (.150)
	**+3**	.010 (.068)	.012 (.068)	t(32) = .494, *p* = .624 (.624)	.-.006 (.154)	.011 (.163)	t(32) = 1.821, *p* = .078 (.150)
**La2**	**-3**	-.023 (.073)	-.021 (.071)	*t*(35) = .700, *p* = .489 (.489)	.011 (.175)	.010 (.166)	*t*(35) = -.121, *p* = .904 (.967)
	**-2**	.014 (.091)	.019 (.088)	*t*(35) = 1.459, *p* = .154 (.216)	.028 (.177)	.027 (.168)	*t*(35) = -.092, *p* = .928 (.967)
	**-1**	.059 (.068)	.067 (.074)	*t*(35) = 1.950, *p* = .059 (.138)	.043 (.192)	.042 (.184)	*t*(35) = -.041, *p* = .967 (.967)
	**0**	.082 (.065)	.095 (.062)	*t*(35) = 3.472, *p* = .001 (.007)	.047 (.196)	.048 (.193)	*t*(35) = .123, *p* = .903 (.967)
	**+1**	.054 (.075)	.067 (.079)	*t*(35) = 3.150, *p* = .003 (.011)	.041 (.197)	.044 (.199)	*t*(35) = .472, *p* = .640 (.967)
	**+2**	.018 (.065)	.024 (.064)	*t*(35) = 1.461, *p* = .153 (.216)	.025 (.183)	.032 (.194)	*t*(35) = .917, *p* = .366 (.967)
	**+3**	.012 (081)	.016 (.084)	*t*(35) = 1.064, *p* = .295 (.344)	.007 (.171)	.016 (.185)	*t*(35) = 1.234, *p* = .226 (.967)

Note. adj = *FDR* adjusted *p*-values. Lags are measured in seconds.
La1 = first joint labyrinth game, La2 = second joint labyrinth game.

Table S11. Results of paired sample t-tests comparing synchrony of actual dyads **after regression of individual and shared positive affect**.

		**IBI synchrony**			**RSA synchrony**		
	**Lag**	**Individual [Mean (SD)]**	**Shared [Mean (SD)]**	**t-test [*t*(df), *p* (*p* adj)]**	**Individual [Mean (SD)]**	**Shared [Mean (SD)]**	**t-test [*t*(df), *p* (*p* adj)]**
**La1**	**-3**				.0822 (.1651)	.1028 (.1739)	t(32) = -1.846, *p* = .074 (.086)
	**-2**	.0328 (.0752)	.0443 (.0765)	t(32) = -2.261, *p* = .031 (.031)	.0779 (.1622)	.1040 (.1668)	t(32) = -2.533, *p* = .016 (.028)
	**-1**	.0437 (.0699)	.0673 (.0769)	t(32) = -5.021, *p* = .000 (.000)	.0663 (.1671)	.0425 (.1916)	t(32) = -3.072, *p* = .004 (.014)
	**0**	.0569 (.0739)	.0761 (.0688)	t(32) = -4.109, *p* = .000 (.000)	.0437 (.1751)	.0756 (.1661)	t(32) = -3.116, *p* = .004 (.014)
	**+1**	.0415 (.0540)	.0540 (.0566)	t(32) = -2.955, *p* = .006 (.010)	.0198 (.1804)	.0492 (.1639)	t(32) = -2.718, *p* = .011 (.026)
	**+2**	.0004 (.0702)	.0088 (.0719)	t(32) = -.2.274, *p* = .030 (.031)	.-.0060 (.1789)	.0172 (.1603)	t(32) = -2.061, *p* = .048 (.067)
	**+3**				-.0227 (.1714)	-.0055 (.1538)	t(32) = -1.472, *p* = .151 (.151)
**La2**	**-3**						
	**-2**	.0035 (.0926)	.0139 (.0908)	t(35) = -1.925, *p* = .062 (.062)	.0050 (.1541)	.0282 (.1767)	t(35) = -1.962, *p* = .058 (.058)
	**-1**	.0428 (.0671)	.0593 (.0682)	t(35) = -3.855, *p* = .000 (.000)	.0129 (.1697)	.0425 (.1916)	t(35) = -2.338, *p* = .025 (.030)
	**0**	.0634 (.0697)	.0823 (.0650)	t(35) = -3.881, *p* = .000 (.000)	.0119 (.1799)	.0471 (.1964)	t(35) = -2.597, *p* = .014 (.021)
	**+1**	.0372 (.0817)	.0540 (.0750)	t(35) = -3.545, *p* = .001 (.002)	.0033 (.1888)	.0412 (.1972)	t(35) = -2.668, *p* = .011 (.021)
	**+2**	.0067 (.0670)	.0184 (.0653)	t(35) = -2.268, *p* = .030 (.038)	-.0128 (.1870)	.0254 (.1830)	t(35) = -2.708, *p* = .010 (.021)
	**+3**				-.0285 (.1851)	.0067 (.1712)	t(35) = -2.639, *p* = .012 (021)

Note. adj = *FDR* adjusted *p*-values. Lags are measured in seconds.
La1 = first joint labyrinth game, La2 = second joint labyrinth game.

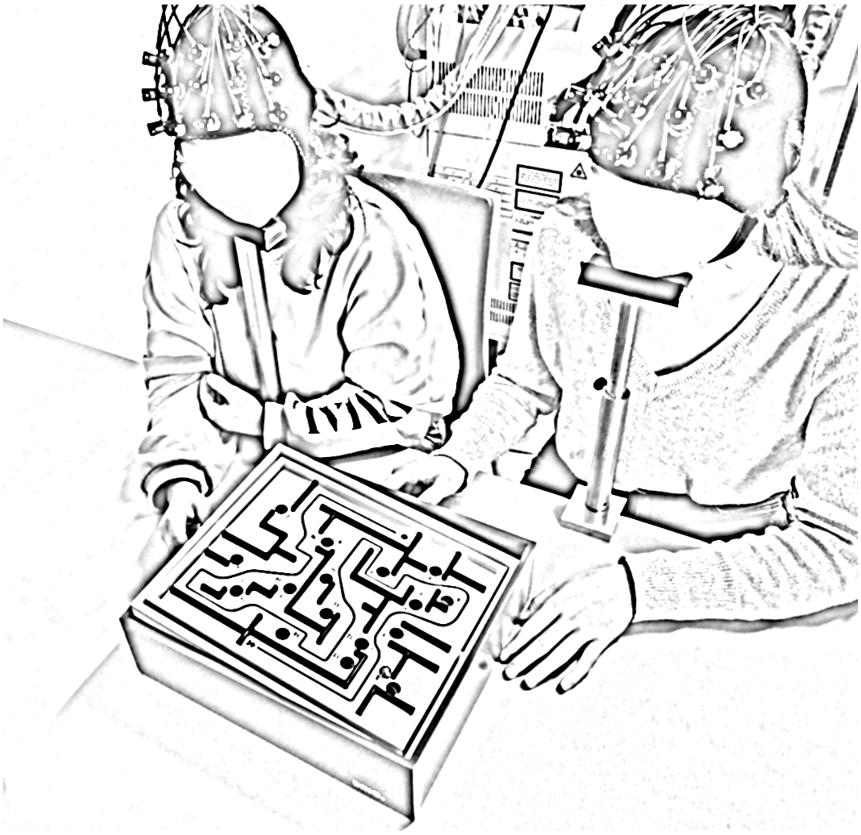

Fig. S1: Experimental set up.

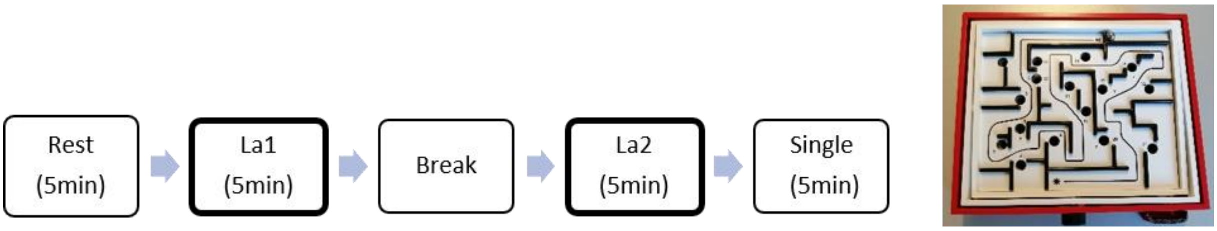

Fig. S2: Left: Illustration of experimental design. After watching a relaxing video for 5 minutes (Rest), the first joint labyrinth game (La1) is played followed by a short break, a second game block of the joint labyrinth game (La2) and finally a single labyrinth game (Single). Right: Picture of the game material.

Category	Code	Content
Body movement	0	No strong body movements
	1	Strong body movements
Affect	0	No affect
	1.1	Negative affect (e.g., frowning, sighing)
	1.2	Positive affect (e.g., laughing, smiling)
Attention	0	Attention on game
	1	Attention on partner
Course of Play	0	No event
	1.1	Event (marble dropped through hole)
	1.2	Event (labyrinth finished successfully)

Fig. S3: Behavioral coding scheme. Mother's and child's behavior is coded on a second-to-second basis.
